# Cytotoxicity and Characterization of Ultrafine Particles from Desktop Three-Dimensional Printers with Multiple Filaments

**DOI:** 10.3390/toxics11090720

**Published:** 2023-08-22

**Authors:** Runcheng Fang, Afzaal Nadeem Mohammed, Jagjit Singh Yadav, Jun Wang

**Affiliations:** 1 Environmental and Industrial Hygiene, Department of Environmental and Public Health Sciences, College of Medicine University of Cincinnati, Cincinnati, OH 45267, USA; runcheng.fang@utah.edu; 2 Environmental Genetics and Molecular Toxicology, Department of Environmental and Public Health Sciences, College of Medicine, University of Cincinnati, Cincinnati, OH 45267, USA; mohamma5@mail.uc.edu (A.N.M.); yadavjs@ucmail.uc.edu (J.S.Y.)

**Keywords:** ultrafine particles, 3D printers, cytotoxicity, air-liquid interface, exposure assessment, HEPA

## Abstract

Previous research has indicated that ultrafine particles (UFPs, particles less than 100 nm) emitted from desktop three-dimensional (3D) printers exhibit cytotoxicity. However, only a limited number of particles from different filaments and their combinations have been tested for cytotoxicity. This study quantified the emissions of UFPs from a commercially available filament extrusion desktop 3D printer using three different filaments, including acrylonitrile butadiene Styrene (ABS), thermoplastic polyurethane (TPU), and polyethylene terephthalate glycol (PETG). In this study, controlled experiments were conducted where the particles emitted were used to expose cells grown in an air-liquid interface (ALI) system. The ALI exposures were utilized for in vitro characterization of particle mixtures, including UFPs from a 3D printer. Additionally, a lactate dehydrogenase (LDH) assay was used to evaluate the cytotoxic effects of these UFPs. A549 cells were exposed at the ALI to UFPs generated by an operational 3D printer for an average of 45 and 90 min. Twenty-four hours post-exposure, the cells were analyzed for percent cytotoxicity in a 24-well ALI insert (LDH assay). UFP exposure resulted in diminished cell viability, as evidenced by significantly increased LDH levels. The findings demonstrate that ABS has the most significant particle emission. ABS was the only filament that showed a significant difference compared to the high efficiency particulate arrestance (HEPA) following 90 min of exposure (*p*-value < 0.05). Both ABS and PETG exhibited a significant difference compared to the HEPA control after 45 min of exposure. A preliminary analysis of potential exposure to these products in a typical environment advises caution when operating multiple printer and filament combinations in poorly ventilated spaces or without combined gas and particle filtration systems.

## 1. Introduction

Commercial desktop three-dimensional (3D) printing technology has grown in popularity in recent years due to its enhanced accessibility, primarily through open-source 3D printers. These devices permit users to print with any suitable material from any supplier. Open-source 3D printers are often favored by hobbyists due to their ease of use and wide array of compatible filaments.

Traditionally, the most popular filaments used in the 3D printer are acrylonitrile butadiene styrene (ABS) and polylactic acid (PLA). However, there have been more developed filaments for 3D printers out there in recent years, including polyethylene terephthalate (PETG), thermoplastic polyurethane (TPU), polyetheretherketone (PEEK), among others.

One of the most widely utilized operating principles of 3D printers is fused deposition modeling (FDM). This technique shapes the object by melting and subsequently cooling the filaments. More specifically, the filaments are melted and directed onto the build platform using a base and nozzle, both controlled by a computer program. The program translates the object’s dimensions into coordinates, guiding the movement of the nozzle and base. As the nozzle traverses the base, the plastic cools and solidifies, forming a complex bond with the preceding layer. Once this process is complete, the printhead elevates to allow for the next layer of plastic to be applied [1].

Three-dimensional printing, also referred to as additive manufacturing, has garnered widespread popularity in various fields due to its capacity to construct intricate structures layer by layer from a digital model. However, this process can result in the emission of ultrafine particles (UFPs) and nanoparticles (NPs), which pose potential health risks, as demonstrated by several research studies [1,2,3]. UFPs—particles with diameters less than 100 nm—and NPs—particles with dimensions on the nanoscale, often within the range of 1–100 nm—can present potential health hazards. Most existing literature investigating the potential health impacts of 3D printing concentrates on the use of polylactic acid (PLA) and other traditional filaments. However, the generation of UFPs and NPs is not confined to these materials alone [1,2,3]. Several in vitro and in vivo studies have indicated that NPs can induce acute lung inflammation [4,5,6,7]. One human case report highlighted that a 28-year-old worker developed work-related asthma ten days after operating ten FDM 3D printers with ABS filaments in a 3000 cubic feet work zone [8].

Air-liquid interface (ALI) systems have recently been developed to address the challenges inherent in studying inhalation exposure with submerged cultures. Many studies indicate that cells exposed to an ALI are significantly more sensitive than those in submerged cultures during in vitro exposure to airborne UFPs. Various studies using in vitro cellular assays with human tumorigenic lung epithelial cells (A549) and in vivo mouse exposure have demonstrated toxic responses upon exposure to PLA- and ABS-emitted particles from an FDM 3D printer [6]. However, few studies to date have explored the emission of multiple filaments with the application of new open-source technology to 3D printers. Even fewer filament materials have been characterized for their toxicity, specifically ABS and PLA [1,2,3,4,5,6,7,8]. Despite prior studies providing insights into the potential health effects of 3D printers, there remain crucial gaps in our understanding of emissions from these devices. Therefore, this study was built upon previous studies by quantifying the emission of particles and using ALI culture models from a commercially available desktop Fused Filament Fabrication (FFF) open-source 3D printer. This printer uses one traditional filament (ABS) and two advanced filaments (PETG and TPU) to print standardized test objects in a test chamber. Results were analyzed to investigate differences in particle emissions and cytotoxicity based on the filament material.

## 2. Materials and Methods

### 2.1. Exposure System Description

**Figure 1:**
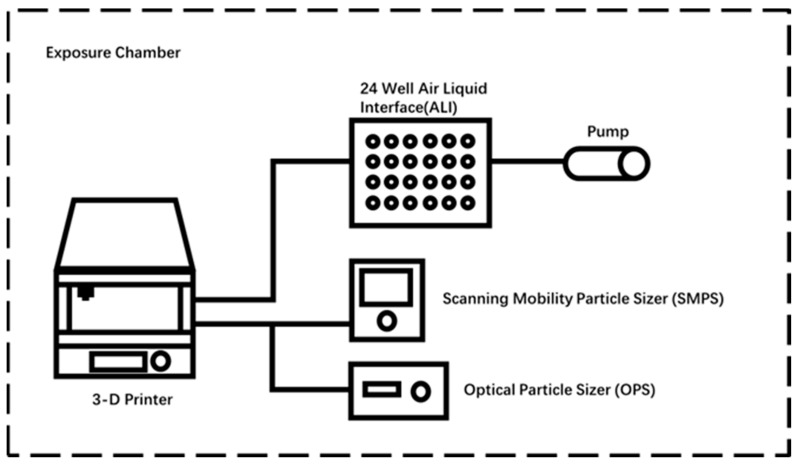
Overview of the exposure system setting.

All measurements were conducted using a single desktop 3D printer with a fume hood (Creator Pro 3D Printer, Zhejiang Flashforge 3D Technology Co., Ltd., Zhejiang, China), placed inside a test chamber. An overview of the experimental setup is depicted in Figure 1. All simulations were carried out at the Workplace Aerosol and Gaseous Laboratory (WANG-LAB) at the University of Cincinnati. To evaluate the distribution of UFPs emitted by 3D printers using multiple filaments, we repeated laboratory simulations three times for each of the three filaments and a blank control in a single 3D printer setup.

The particles collected were measured inside the tube connecting the 3D printer to the ALI system. These particles were characterized using a scanning Mobility Particle Sizer (SMPS) (Model 3910, TSI Inc., Shoreview, MN, USA) for 0–420 nanometer (nm) aerosol, and an Optical Particle Sizer (OPS) (Model 3330, TSI, Shoreview, MN, USA) for 0.4–10 micron (μm) aerosol measurements. These measurements illustrate the aerosol exposure of pollutants generated by 3D printers using different filaments in the same working environment. The generation and sampling of 3D printer particles were carried out based on previous studies [9,10,11,12,13]. The tested 3D printer was equipped with a plastic cover provided by the manufacturer, which rests atop the device but does not form a tight seal. The printer was connected via a tube to the instruments, as shown in Figure 1.

**Figure 2:**
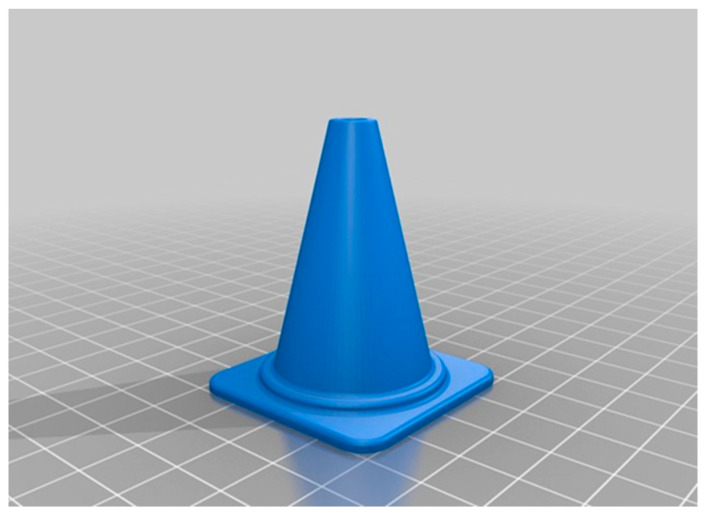
Printed traffic cone.

Even though the manufacturer recommended different extrusion temperatures for the various feedstocks, we standardized the temperature to neutralize it as a variable and eliminate its effect on aerosol generation; the filament extruder was set at 220 °C [9]. The parameters for the experimental conditions are enumerated in Table 1. Simultaneously, specialized tubing interfaced the operational 3D printer with the air-liquid interface, utilizing technology provided by CH Technologies Inc. (Westwood, NJ, USA). High-throughput pumps, situated externally to the system, governed the flow within the exposure systems at a precisely controlled rate of 0.6 L per minute (lpm), implemented through an in vitro methodology. For all tests, except the blank tests, we printed a 40 × 40 × 50 mm traffic cone sample from the Creator Pro 3D Printer, as illustrated in Figure 2.

### 2.2. Aerosol Measurement

A scanning mobility particle sizer (SMPS, TSI 3910 NanoScan, Minneapolis, MN, USA) was utilized to record aerosol data during preheating, extrusion, printing, and five minutes post-extrusion, observing the decay in aerosol number concentration within the chamber. Each filament underwent three runs. The SMPS facilitated the real-time measurement of nanoparticles in size ranges of 10–420 nm. The SMPS employs isopropyl alcohol within its internal condensation particle counter (CPC) to precisely record aerosolized concentrations ranging from low to high (1,000,000 particles/cm^3^). Its sixteen size channels allow researchers to measure particle sizes from 0.3 to 10 μm. The SMPS 3910 sampled at a flow rate of 0.6 lpm. For the extruder heating element preset at 220 °C, the sidewall temperature of the extruder reaches 220 °C after an average heating period of 5 min, initiating the extrusion. The filament extruder operated at 220 °C for three different raw materials. Data were recorded using SMPS 3910 during the extrusion and continued for five minutes after the extrusion had ceased, to observe the decay in particle number concentration within the chamber.

The particle number concentration data from each run for each plastic type were analyzed. Concentration data measured by the SMPS were averaged for each plastic type, and a standard deviation at each point was calculated.

### 2.3. Cytotoxicity Assessments

In this study, A549 cells, a type of human lung epithelial cell line obtained from the American Type Culture Collection (ATCC), were used for cytotoxicity assessment. The cells were cultured in T25 flasks using F-12K Medium, supplemented with 10% fetal bovine serum and 1% penicillin-streptomycin for optimal growth conditions. Cells were passaged twice before plating on 24-well Transwell inserts (0.4-µm pore size) for ALI. These inserts, typically integrated into multi-well plates, provide a porous surface for cell growth. This system was chosen to ensure a more physiologically relevant environment that mimics in vivo conditions where cells are exposed to the air on one side. After the second passage, cells were trypsinized and seeded at 1 × 10^5^ cells/insert, then incubated for 4 h for attachment. The media was carefully removed from both apical and basal chambers, and 125 µL of fresh medium was added to the basal chamber. Cells were then allowed to maintain in the ALI settings for 24 h at 37 °C with 5% CO_2_ before transportation to the exposure chamber.

A549 cells grown on the inserts were exposed to particles emitted during the 3D printing sessions, using different printing materials, for an average of 45 or 90 min durations. After exposure, ALI cultures were incubated at 37 °C for 24 h and analyzed for percent cytotoxicity using CYQUANT LDH Cytotoxicity Assay (Invitrogen, Waltham, MA) (n = 3 for each exposure). Lactate dehydrogenase (LDH) is an enzyme present in the cytoplasm which is released outside of the cells upon damage to the cell membrane.

To calculate the percent cytotoxicity, maximum LDH activity and spontaneous LDH activity were determined in cells in the upper chamber and medium in the lower chamber of Transwells not exposed to 3D printing sessions. LDH release was determined in the basal medium of Transwells exposed to each printing session. LDH release and maximum LDH activity were normalized by subtracting the spontaneous LDH activity. Percent cytotoxicity was calculated by the ratio of normalized LDH release to maximum LDH activity.

### 2.4. Statistical Analysis

Statistical analysis of the data collected from the simulated chemical exposure events was conducted using Microsoft Excel^®^. T-tests were used to compare the results of two different groups at an alpha of 0.05. Statistical significance between the cytotoxicities among the different groups was calculated using one-way analysis of variance (ANOVA) models with Tukey’s post hoc multiple comparisons. A *p*-value of less than or equal to 0.05 was considered statistically significant.

## 3. Results and Discussion

### 3.1. Aerosol Measurements

The description statistic of all tests is presented in Table 2. The printing times varied depending on the type of filament used. Of all the filaments evaluated, TPU had a peak particle number concentration of 540,033.3 #/cm^3^, PETG had 1,884,866.7 #/cm^3^, while ABS recorded the highest at 2,572,400 #/cm^3^.

By evaluating the particle number concentration data derived from each run of each type of plastic, we found that particle concentration varied across the different filament types. The particle concentration measurements obtained using the SMPS were averaged for each type of plastic, and the standard deviation at each point was calculated to reflect the variability of the particle concentration for each filament type.

**Figure 3:**
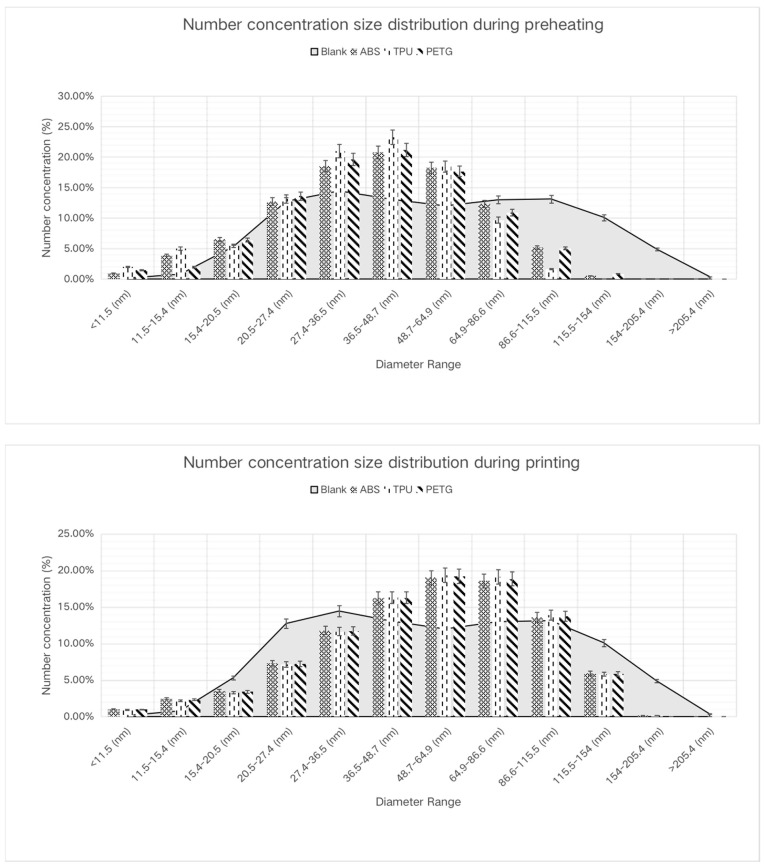
Particle size distribution in different print phases: preheating (**above**) and printing (**below**).

The size distribution data extracted from each test run provided insightful findings. Our graphical representation of these data (Figure 3) compares the particle size distribution for ABS, PETG, and TPU filament printing during preheating and printing tasks with the blank. The graph illustrates that the number of particles with a diameter less than 46.7 nm was significantly higher during the preheating task than in the printing phase, indicating the emission of smaller particles during preheating.

The concentration of all particulate matter generated by filaments with dimensions less than 48.7 nm was significantly elevated during the initial preheating phase of the 3D printing process, in sharp contrast to baseline levels. In contrast, particle concentrations between 48.7 nm and 64.9 nm in diameter resembled background concentrations.

The measured particle number concentration, however, remained lower than the background concentration throughout the preheating stage, and this difference was more evident with an increase in particle dimension. A critical diameter of 64.9 nm caused this divergence to start.

Following preheating, the incidence of particles larger than 46.7 nm in diameter increased significantly during the printing phase involving ABS and TPU materials as compared to the preheating phase. This difference becomes more pronounced as particle diameter increases. At the 46.7 nm threshold, PETG particles had a somewhat greater occurrence than ABS and TPU; however, this difference became more pronounced for particulates larger than 64.9 nm in diameter.

The particle number concentrations linked with the three filament types used in the printing process exhibited a wide range of distributions. ABS, TPU, and PETG concentrations were mostly concentrated within a diameter range of 36.5–115.5 nm, with particle counts exceeding background values in this spectrum. This concentration was mostly found between the scanning diameters of 48.7–64.9 nm and 64.9–86.6 nm.

**Figure 4:**
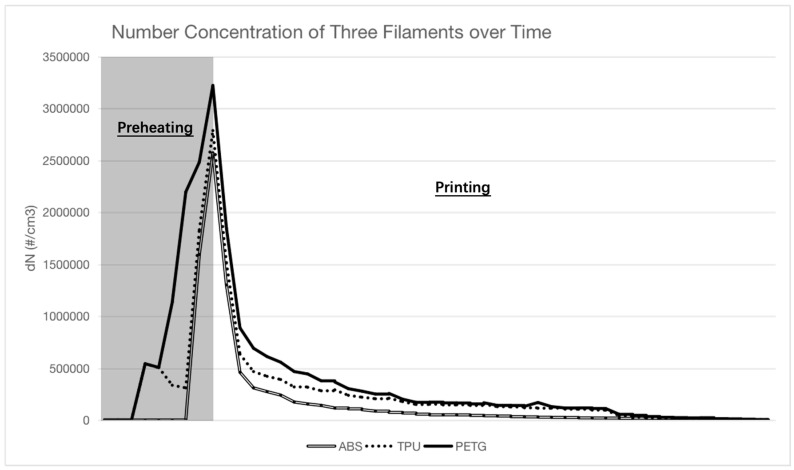
Particle number concentration of three different filaments over time.

We used SMPS measurements to follow and understand the variations in total particle number concentration during the various stages of the 3D printing process. These measurements enabled real-time monitoring of particle concentrations throughout the technique, from background level assessment to the preheating and printing phases, culminating in the cooling phase (Figure 4). The observed operation patterns for the three filament types—ABS, TPU, and PETG—were analogous and may be classified into three separate operational stages: preheating, printing, and cooling. The preheating phase was characterized by a substantial increase in particle number concentration, which reached its peak. Following that, a significant decrease in particle number was seen during the printing step, with concentrations dropping to near-background levels throughout the cooling phase.

In Table 3, the aerosol particle number size distribution for the three different types of filament (ABS, TPU, and PETG) and the control (blank) combustion process is shown. The average total particle number concentration produced by ABS was calculated by empirical study to be 155,056.46 particles/cm^3^. The central tendency of this distribution was determined to have a geometric mean (GM) of 66.01 nm. A polydisperse size distribution, referred to as a distribution covering a wide range of particle sizes, is indicated by a geo-metric standard deviation (GSD) of 1.75. Data in aerosol studies usually follow log-normal distributions. Therefore, as compared to conventional statistical measurements, geometric parameters like GM and GSD provide a more realistic characterization of these distributions. The GSD offers a practical measurement of size diversity within the distribution, but the GM delivers a precise estimate of the ‘average’ particle size. Since our aerosol data are log-normal, this geometric approach provides the most helpful information. The TPU, PETG, and blank descriptions are concurrently condensed in Table 3. Notably, ABS is the only filament with a GM greater than the blank when compared to the blank.

### 3.2. Cytotoxicity Assessments

**Figure 5:**
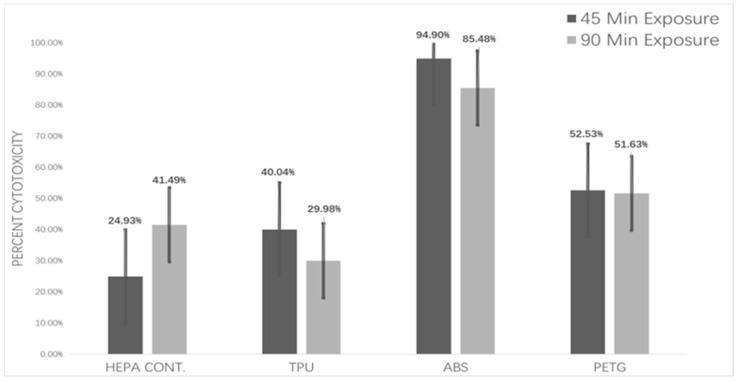
Percent cytotoxicity in A549 cells grown on air-liquid interface (ALI) exposed to particles emitted from working 3D printer.

In this study, human alveolar epithelial type II A549 cells were cultivated under ALI conditions and exposed for 45 and 90 min to UFPs produced by a working 3D printer. Figure 5 shows the results of an LDH experiment used to evaluate the cytotoxicity brought on by this exposure. The percentage of LDH released into the basal chamber, which represents cell damage and lysis, was used to calculate the percentage of cytotoxicity. According to our research, cytotoxicity increased with a 45 min exposure time as opposed to a 90 min exposure. Further research is necessary to see whether there are any potential long-term adaptive cellular responses to this seemingly paradoxical conclusion.

In terms of filament types, the one-way ANOVA results outlined in Table 4 revealed that both ABS and PETG demonstrated significant deviations in their cytotoxic profiles compared to the HEPA filter control group, following a 45 min exposure duration. ABS, in particular, demonstrated the highest cytotoxicity among all groups, and it maintained this significantly different cytotoxic profile relative to the HEPA control group even after 90 min of exposure (*p*-value < 0.05).

### 3.3. Health Implications

The data showed significant differences in particle number concentration across the filament types ABS, TPU, and PETG, indicating the distinct aerosol properties of different 3D printer filaments. ABS displayed the highest particle concentration of 2.6 × 10^6^ #/cm^3^, which is similar to the previous study which reported that concentrations were up to 10^6^ #/cm^3^ when printing with ABS [1,2,3]. This finding of ABS emission aligns with the material properties of ABS, which is created by polymerizing acrylonitrile and styrene in the presence of polybutadiene. The polymerization process can generate a large number of tiny particles, leading to a high concentration. Additionally, the higher geometric mean for ABS could be a result of remnants of the material in the print head that do not melt, leading to potential blockages and the inconsistent emission of particles. In contrast, PETG and TPU, which are produced using different processes, demonstrated lower particle concentrations emitted during printing. PETG is made through a two-step melt-phase polycondensation process, and TPU is derived from tetrahydrofuran ethers. These processes could potentially lead to fewer particle emissions, though further research is required to confirm this assumption. It is important to examine particle emissions across various filament types. Prior studies predominantly focused on PLA and ABS filaments, leaving a knowledge gap regarding other commonly used materials like TPU and PETG which were used in applications similar to 3D printing [14,15,16]. By exploring these materials, we’ve been able to demonstrate the variation in particle emissions, enabling better understanding of the potential health implications linked to different filaments. Three-dimensional printing users need to avoid printing with filaments emitting high concentrations of ultrafine particles.

While particles were consistently emitted during each test, a mean particle diameter of 50–60 nm confirmed the majority of the particles emitted were ultrafine particles. A marked increase in emissions from the 3D printer was observed as printing began with all filaments, consistent with the emissions profile outlined in earlier studies [1,2,3,4,5,6].

It is important to understand the cytotoxicity of these particles in addition to their physical and chemical properties. We established that A549 cells, grown in an air-liquid interface (ALI), can be successfully exposed to UFPs produced by a working 3D printer. Our cytotoxicity assessments found that both short (45 min) and long (90 min) exposures increased cytotoxicity compared to HEPA controls, highlighting potential health risks. It was particularly notable that ABS showed the highest cytotoxicity during both exposure times.

## 4. Conclusions

This study analyzed and compared the aerosol emissions and cytotoxicity assessments of three different types of 3D printer filaments: ABS, TPU, and PETG. The findings of the study present insights into the aerosol emission profiles of these filaments and the potential health impacts. From the aerosol measurement results, it was evident that different types of filaments produce varying particle concentrations. The ABS filament showed the highest concentration of ultrafine particles, whereas TPU and PETG filaments showed lower concentrations. These disparities are likely due to the distinct material compositions and thermal properties.

In addition, the study also found that the particle emissions of the 3D printer varied across different operational phases. The preheating phase was particularly notable, with particle concentrations reaching their peak before subsequently decreasing during the printing phase. This highlights the need for the implementation of safety measures during the 3D printing process, particularly during the preheating phase.

The cytotoxicity assessments further underlined the potential health hazards associated with UFPs exposure. Shorter exposure durations resulted in higher cytotoxicity in this study, indicating the importance of exposure time to 3D printer emissions. The ABS filament displayed the highest cytotoxicity, making it crucial to take necessary precautions while using this filament for 3D printing.

Further research is required to understand the health implications of these particulate and gaseous emissions. Potential future studies could focus on exploring the health effects of long-term exposure to these particles, the impact of varying printing parameters, and the effectiveness of various safety measures.

## Figures and Tables

**Table 1:** Experimental conditions of different filaments.

Filaments	Extruder Temperature (°C)	Bed Temperature (°C)
Blank	24	24
ABS	220	85
TPU	220	50
PETG	220	24

**Table 2:** Descriptive statistics of particle number concentration from printing with different filaments.

Filaments	Median	Mean	Min, Max	Std. Deviation	*p*-Value
Blank	1002.2	1002.2	1002.2, 1002.2	0	N/A
ABS	25,898.3	155,056.5	1754.5, 2.572 × 10^6^	433,084.5	<0.001
TPU	15,879.2	117,212.3	956.1, 540,033.3	116,981.3	<0.001
PETG	95,525.8	124,563.0	950.7, 1.885 × 10^6^	306,704.8	<0.001

**Table 3:** Average geometric mean (GM), geometric standard deviation (GSD) and mean diameters (Mean) (nm) of emitted particles.

Filaments	GM	GSD	Mean
Blank	62.94	1.93	77.65
ABS	66.01	1.75	75.99
TPU	60.90	1.77	70.65
PETG	49.89	1.75	57.81

**Table 4:** ANOVA result: Single factor of HEPA control.

	Filaments	F	*p*-Value	df
	ABS	289.90	2.62 × 10^−6^ *	7
45 min	TPU	2.60	0.18	5
	PETG	7.37	0.042 *	6
	ABS	30.70	0.005 *	5
90 min	TPU	2.10	0.22	5
	PETG	0.32	0.60	5

* *p*-value < 0.05.

## Data Availability

The data that support the findings of this study are available from the corresponding author, J.W., upon reasonable request.
